# Effect of Alkalization With Potassium Carbonate and Ammonium Bicarbonate on the Chemical and Physical Properties of Cocoa Butter: A Factorial and Central Composite Design Approach

**DOI:** 10.1002/fsn3.72469

**Published:** 2026-10-13

**Authors:** Afghan Sadeghzadeh, Maryam Taghinezhad, Sajad Pirsa

**Affiliations:** ^1^ Afagh Higher Education Institute of Urmia Urmia Iran; ^2^ Department of Food Science and Technology, Faculty of Agriculture Urmia University Urmia Iran

**Keywords:** alkalization, ammonium bicarbonate, cocoa butter, physical and chemical properties, potassium carbonate

## Abstract

This study investigated the effects of cocoa butter alkalization using potassium carbonate and ammonium bicarbonate (0%–6%) on acidity, peroxide value, iodine value, saponification value, moisture content, slip melting point, pH, and lead content. A factorial design and central composite design (CCD) were employed. The highest peroxide value (0.1 meq O_2_/kg) was observed in samples containing 3% potassium carbonate +3% ammonium bicarbonate and 6% potassium carbonate +6% ammonium bicarbonate. The highest acidity was related to the control sample (1.77), while the lowest was found in the sample with 6% of both alkalis (0.77). The control sample also showed the lowest saponification value (189.61 mg KOH/g fat), whereas the highest was recorded for the 6% + 6% sample (199.04 mg KOH/g fat). The highest iodine value (35.91 g I_2_/100 g fat) belonged to the 6% + 6% sample, and the lowest (32.42 g I_2_/100 g fat) to the sample containing 6% ammonium bicarbonate alone. Minimum moisture content (0.053%) was observed in the sample with 6% potassium carbonate +3% ammonium bicarbonate, and maximum (0.077%) in the sample with 3% + 3%. The lowest pH (5.05) was for the control, and the highest (9.87) for the 6% + 6% sample. The highest slip melting point (30.23°C) was recorded for the control, and the lowest (26°C) for the sample with 3% potassium carbonate +6% ammonium bicarbonate. All samples complied with Iranian national standards for lead content. The results indicated that 3% potassium carbonate alone, 3% ammonium bicarbonate alone, and 3% potassium carbonate +6% ammonium bicarbonate are suitable for cocoa alkalization. Finally, mathematical models were developed using response surface methodology to establish quantitative relationships between the chemical properties of cocoa butter and the concentrations of the alkaline agents, enabling the achievement of desired product characteristics.

## Graphical abstract

Schematic representation of the alkalization of cocoa butter with potassium carbonate and ammonium bicarbonate and its effects on physicochemical properties including acidity, pH, saponification value, iodine value, moisture content, slip melting point, peroxide value, and lead content.

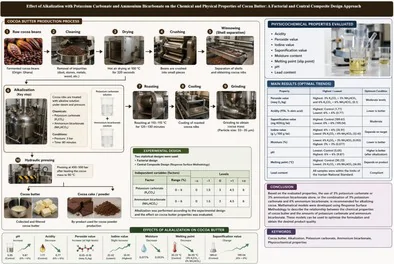

## 1 Introduction

Cocoa beans are the fruits of the cocoa tree and are widely cultivated in tropical and equatorial regions primarily for the extraction of cocoa butter and cocoa powder. Cocoa has three main varieties: Forastero, Criollo, and Trinitario, among which the Forastero variety is the most commonly cultivated type in Central and West Africa (Goude et al. 2023). The cocoa bean consists of 15% husk and 85% kernel, and due to its specific fatty acid and triacylglycerol composition, it is suitable for chocolate production. Cocoa beans typically contain 50%–55% fat, known as cocoa butter, which is used in food, cosmetic, pharmaceutical, and chemical products (Mounjouenpou et al. 2018). Cocoa butter is one of the most fundamental ingredients in chocolate formulations, contributing to blood pressure reduction and cholesterol regulation. Moreover, it is cholesterol‐free and, owing to the presence of certain flavonoids and antioxidants such as tocopherols, supports cardiovascular health and plays a role in cancer prevention. Due to its moisturizing properties and a melting point close to body temperature, cocoa butter is also used in the production of lotions and skin soaps, as it is readily absorbed by the body. According to global statistics, the demand for cocoa and its derived products is increasing, such that worldwide cocoa production approximately doubled from 2008 to 2016 (Voora et al. 2019).

Therefore, a better and more comprehensive understanding of the characteristics and properties of cocoa beans and the butter derived from them can help improve the quality of the resulting products. In the production process of cocoa‐based products, the continuous phase of adding cocoa butter is the sole factor that determines the dispersibility of the constituent compounds, the physical behavior of the product, its appearance, and its distinctive aroma and taste. Cocoa butter, the fat obtained from cocoa tree seeds, is considered one of the most valuable vegetable fats in the chocolate industry. According to global statistics, due to increasing demand, especially in chocolate production, the production rate of cocoa butter is rising, and the production and quality of cocoa‐based products depend on the physical and chemical properties and the constituent compounds of cocoa butter (Kongor and Rahadian Aji Muhammad 2024). In the alkalization process, the beans are automatically received and cleaned by a machine, where all foreign materials are removed. After passing through the cleaning and drying stages, the beans enter a peeling machine, where the husk is separated from the kernel. Subsequently, for roasting, the kernels are introduced into a roaster, and during roasting, alkalization is carried out using alkaline solutions. Following this stage, the kernels are cooled, ground to reduce particle size, converted into cocoa liquor, and finally subjected to pressing to obtain cocoa butter (Valverde Garcia et al. 2020).

Cocoa butter has a relatively simpler triacylglycerol composition compared to other vegetable fats; however, minor changes in this composition can affect its physicochemical properties and ultimately alter its behavioral characteristics. The compounds present in cocoa butter include two categories: glyceridic and non‐glyceridic compounds. The main glyceridic composition of cocoa butter consists of triacylglycerols containing palmitic, oleic, stearic, and linoleic acids, and most of the triacylglycerols are symmetrical, giving rise to the unique properties of cocoa butter. The non‐glyceridic compounds, which constitute a minor fraction of cocoa butter, include four types of proteins (albumin, glutelin, prolamin, and globulin), sterol and triterpene compounds, phospholipid compounds, and carbohydrates. The protein content also varies under the influence of climatic conditions. Therefore, understanding the properties of cocoa butter is critically important for producing chocolates of desirable quality (Colella et al. 2023).

Alkalization of cocoa butter, as opposed to cocoa powder, is of particular interest because it directly affects the fat phase, influencing properties such as acidity, free fatty acid content, oxidative stability, and melting behavior. These parameters are critical determinants of cocoa butter functionality in chocolate manufacturing, affecting texture, mouthfeel, and product stability. While alkalization of cocoa powder has been extensively studied, the direct application to cocoa butter and its specific effects on fat quality have received limited attention.

Accordingly, various studies have been conducted on the preparation and properties of cocoa butter as well as the dynamic changes of its triglyceride compounds during crystallization (Salminen et al. 2020). The transition of cocoa butter into the secondary crystallization stage alters the crystal structure from spherulitic to acicular, leading to changes in polymer morphology and ultimately its rheology, which can modify the characteristics of the final product (Yao et al. 2020). Hence, understanding these properties is of particular importance for producing high‐quality products.

Potassium carbonate was used as a dough leavening agent before the advent of baking powder. This substance has long been used for water softening. Today, it is also employed to reduce the acidity of food products because, when dissolved in water, it makes the solution strongly alkaline. This compound is essential for cocoa alkalization, enhancing color and flavor, reducing bitterness, lowering fat content, and improving its miscibility with liquids (Neeharika et al. 2020).

Potassium carbonate and ammonium bicarbonate were selected for this study based on the following considerations: (i) potassium carbonate is widely used in commercial cocoa alkalization for its effectiveness in pH adjustment and color development (Neeharika et al. 2020); (ii) ammonium bicarbonate, while commonly used in baking applications, has not been systematically studied for cocoa butter alkalization, offering a novel perspective; and (iii) both compounds leave no toxic residues (potassium and ammonium are Generally Recognized as Safe—GRAS), making them suitable for food applications. The comparison between these two agents with distinct chemical properties (carbonate vs. bicarbonate) allows for systematic evaluation of their differential effects on cocoa butter.

Ammonium bicarbonate is also used as a leavening agent in dough for preparing various baked goods. It produces a fluffy dough that is highly suitable for making breads, crackers, and certain cookies. Ammonium bicarbonate is typically used in small baked goods because it can rapidly disperse and evaporate ammonia gas within the baked product. Although an ammonia aroma may be present during the baking process, it does not persist upon consumption and is not noticeable in the final baked products (Sun et al. 2024). The use of ammonium bicarbonate in baked goods results in a light, crispy, and somewhat soft texture. Although this compound is nowadays often replaced by baking powder or baking soda, its use is still preferred by those who desire a lighter or crispier texture. This compound becomes active when added to the dough or batter mixture and is heated or baked at room temperature, as heat is the most important factor in activating the chemical reaction and producing gases that cause the baked goods to rise (Yao et al. 2020). Anhydrous ammonia is used in synthetic food colors for energy drinks, spices, breakfast cereals, ice cream and sorbet, jellies, chips, puffed snacks, sauces, canned products, etc. In the cosmetic and hygiene industry, this compound is used to balance the pH of the reaction medium and as a buffering agent.

Cocoa butter has a relatively simpler composition compared to other vegetable fats, but its properties and constituent compounds are strongly influenced by environmental and geographical factors. The unique triglyceride composition of cocoa butter and its low diglyceride content impart desirable properties to it, enabling recrystallization during various chocolate manufacturing processes (Talbot 2017).

The novelty of the present study lies in three aspects: (i) while cocoa powder alkalization is well‐documented, the systematic investigation of alkalization effects on cocoa butter is scarce; (ii) to the best of our knowledge, this is the first study to compare potassium carbonate and ammonium bicarbonate directly on cocoa butter using a central composite design; and (iii) the development of predictive mathematical models for multiple physicochemical parameters of cocoa butter under alkalization conditions has not been previously reported. Therefore, the aim of the present study is to investigate the effect of potassium carbonate and ammonium bicarbonate on the physicochemical properties of cocoa butter. Understanding these effects is important because cocoa butter quality directly influences chocolate texture, stability, and sensory attributes (Talbot 2017). By establishing quantitative relationships between alkalization conditions and cocoa butter properties, the study aims to provide process optimization tools for the chocolate industry. The term “quality” in this context refers to specific, measurable parameters: reduced acidity, controlled oxidative stability, appropriate melting behavior, and compliance with food safety standards.

## 2 Materials and Methods

### 2.1 Chemicals and Equipment

In this study, fermented cocoa beans (obtained from Ghana), potassium carbonate (purchased from Altair Chemica, Italy), and ammonium bicarbonate were used. The chemicals, including hexane, sodium hydroxide, phenolphthalein, ethyl alcohol, acetic acid, Hanus reagent, starch paste, and other chemical compounds, were procured from Merck (Germany) and used without further purification. A bean cleaner, dryer, grinder, vibrating sieve, and winnowing machine (all manufactured by Lehmann, Germany) were employed. A mixing tank for potassium carbonate and water, a mixer, a roaster, and a cooling unit (all manufactured by Bühler, Germany) were used. The storage tank (manufactured locally in Iran), cocoa liquor press, and balance (manufactured by Carlo Montanari, Italy) were utilized. A pH meter (Metrohm, Switzerland), an electric furnace (Atash, Iran), a balance model PX 3000 (Germany), an oven (Vargha Tajhiz, Iran), and an atomic absorption spectrophotometer model 220Z (Varian) were used in this study.

### 2.2 Preparation of Cocoa Butter

Fermented cocoa beans (Ghana origin) were processed at Shirin Asal Company (Tabriz, Iran). The preparation followed these sequential steps:

Step 1—Cleaning: Beans were cleaned using vibrating screens (with holes of different diameters: 4 mm, 6 mm, and 8 mm) to remove foreign materials larger or smaller than the beans. Air screens removed low‐density impurities, and magnets extracted iron particles.

Step 2—Drying: The cleaned beans were dried in a hot air dryer at 100°C for 320 s to facilitate shell separation.

Step 3—Crushing and Separation: Beans were passed through a disc‐type grinder (Lehmann, Germany) to crack them, followed by separation of shells from nibs using vibrating screens and air flow (light shells were blown away, while heavy nibs passed through).

Step 4—Alkalization: Nibs were treated with alkaline solutions (potassium carbonate and/or ammonium bicarbonate) in a mixing tank (Bühler, Germany) under steam at 3 bar for 80 min.

Step 5—Roasting: The alkalized nibs were roasted in a drum roaster (Bühler, Germany) at 110°C–115°C for 125–130 min, reducing moisture content to 1.5%–2%.

Step 6—Cooling and Milling: The roasted nibs were cooled and milled to achieve a particle size of 33–35 μm.

Step 7—Pressing: The cocoa liquor was heated to 90°C, homogenized, and pressed in a pot‐type press (Carlo Montanari, Italy) at 450–500 bar to separate cocoa butter from cocoa powder (Figure 1).

**FIGURE 1:**
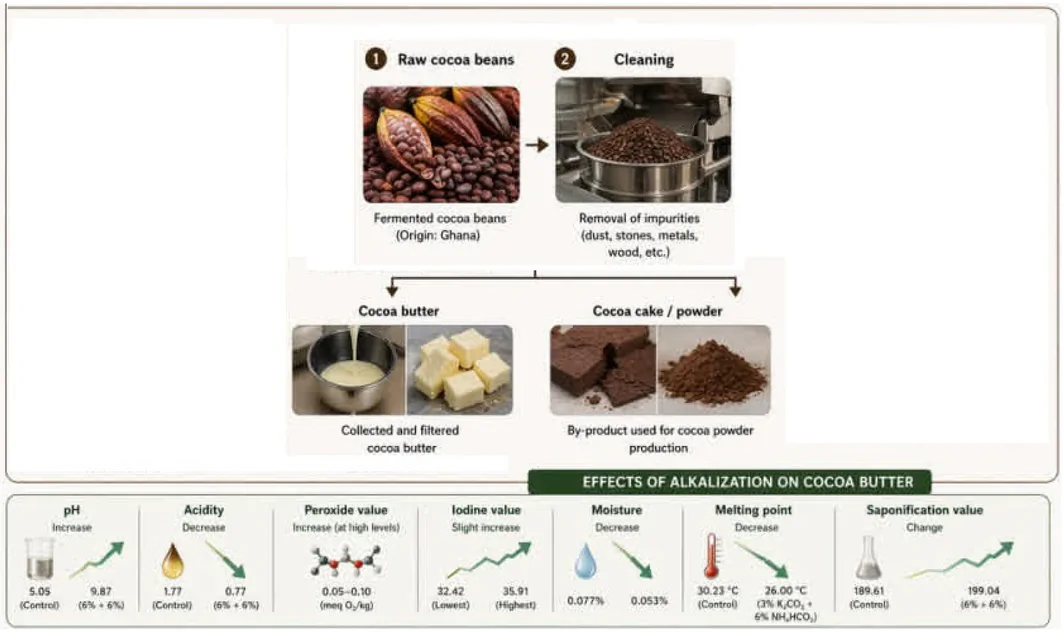
Scheme of preparation of cocoa butter and its tests.

The prepared treatments in this study and the amounts of potassium carbonate and ammonium bicarbonate added for cocoa powder production followed the statistical design (Table 1).

**TABLE 1:** List of experiments designed according to the central composite design.

Run	Factor A: K_2_CO_3_ (%)	Factor B: NH_4_HCO_3_ (%)
1	6	0
2	3	3
3	6	6
4	3	3
5	0	6
6	3	3
7	0	0
8	3	3
9	3	0
10	3	6
11	0	3
12	6	3
13	3	3

### 2.3 Tests Performed on Cocoa Butter

#### 2.3.1 Acidity

The acidity of the samples was determined according to the method described in Iranian National Standard No. 608 (ISIRI 2011). For this test, 2.5 to 3.5 g of cocoa butter fat was mixed with 50 mL of hot neutralized ethyl alcohol and a few drops of phenolphthalein indicator, and then titrated with standard sodium hydroxide. The acidity of cocoa butter was calculated using Equation (1):

(1) $$\text{Acidity}\;\left(\%\right)=\frac{28.2\times V\times N}{\text{Sample weight}}\times 100$$

*V:* Volume of standard sodium or potassium hydroxide solution used in titration (mL). *N*: Normality of standard sodium or potassium hydroxide solution.

#### 2.3.2 Peroxide Value

The peroxide value of the samples was determined according to the method described in Iranian National Standard No. 608 (ISIRI 2011). For this test, nitrogen gas was first passed through a thoroughly cleaned Erlenmeyer flask. Then, 5 g of the test sample (with an accuracy of 0.1 mg) was weighed into the flask and rinsed with a solution of glacial acetic acid in isooctane to ensure that the flask contained no oxidizing or reducing substances. Subsequently, 0.5 mL of saturated potassium iodide solution was added. The flask was sealed and mixed with a magnetic stirrer without vigorous rotation for 60 s. The flask was then opened, and 100 mL of freshly boiled distilled water was immediately added. The liberated iodine was promptly titrated with a few milliliters of 0.01 N standard sodium thiosulfate solution until the color changed from orange‐yellow to pale yellow. Then, 0.5 mL of starch solution was added, producing a purple color, which was then titrated until colorless. The titration was considered complete when the colorless solution remained stable for 30 s. A blank sample was titrated simultaneously. The peroxide value, expressed as milliequivalents of active oxygen per kilogram, was calculated using Equation (2):

(2) $$\mathrm{pv}=\frac{\left(V-V_{0}\right)\times C\times F}{m}\times 100$$

*V*: Volume of sodium thiosulfate solution used for the sample (mL). *V*_0_: Volume of standard sodium thiosulfate solution used for the blank (mL). *C*: Concentration of sodium thiosulfate solution (mol/L). *m*: Mass of the test portion (g). *F*: Factor of 0.01 N sodium thiosulfate solution.

#### 2.3.3 Saponification Value

The saponification value was measured according to the method described in Iranian National Standard No. 6082 (ISIRI 2012). For this test, 5 g of the test sample was weighed into an Erlenmeyer flask, and 50 mL of alcoholic potassium hydroxide solution was added. The flask was connected to a condenser and heated in a hot water bath for 30 min, ensuring that the mixture did not stop boiling during this period. When the solution became completely homogeneous, saponification was complete. The condenser was then rinsed with distilled water, and the contents were cooled. Subsequently, 1 mL of phenolphthalein indicator was added, and the excess potassium hydroxide that had not participated in the saponification reaction was titrated with 0.5 N hydrochloric acid. All steps were also performed for a blank sample. The saponification value was calculated using Equation (3):

(3) $$\mathrm{Sap}\;\text{value}\;\left(\text{mgKOH}/100\;g\right)=\frac{V_{s}-V_{0}}{\text{Sample weight}}\times N\times 56.1$$

*V*_s_: Volume of HCl for the sample (mL). *V*_0_: Volume of HCl for the blank (mL).

#### 2.3.4 Iodine Value

The iodine value was measured according to the method described in Iranian National Standard No. 4886 using Hanus solution (ISIRI 2013). For this test, 0.25 g of the sample was weighed into a ground‐glass stoppered Erlenmeyer flask, and 10 mL of chloroform was added. Then, 25 mL of Hanus reagent was added using a bulb pipette, and the flask was placed in a dark place for 30 min. Subsequently, 15 mL of 15% potassium iodide solution was added, followed by 100 mL of boiled and cooled distilled water. The solution was then titrated with 0.1 N sodium thiosulfate solution until the reddish‐brown color turned yellow. Then, 10 drops of starch paste were added, and titration was continued until complete decolorization. The volume consumed for the sample was the sum of two titrations. The iodine value was calculated using Equation (4):

(4) $$I\;\text{value}\;\left(g/100\;g\right)=\frac{V_{1}-V_{2}}{\text{Sample weight}}\times N\times 12.69$$

*V*_1_: Volume of sodium thiosulfate solution consumed for the blank (mL). *V*_2_: Volume of sodium thiosulfate solution consumed for the sample (mL). *N*: Normality of sodium thiosulfate solution.

#### 2.3.5 Moisture Content

Moisture content was determined according to Iranian National Standard No. 4291 (ISIRI 1998). For this purpose, a specified amount of the sample (5 g) was weighed and placed in an oven at 105°C. After achieving constant weight, the samples were reweighed, and the moisture content was calculated using Equation (5):

(5) $$\text{Moisture}\;\left(\%\right)=\frac{M_{i}-M_{f}}{M_{i}}\times 100$$

*Mᵢ*: Initial weight of the test sample (g). *M*_*f*_: Weight of the test sample after oven drying (g).

#### 2.3.6 Slip Melting Point (SMP)

The slip melting point (SMP) was measured according to the open capillary tube method AOCS Cc 3–25 (AOCS 2017). The samples were liquefied at 10°C above their melting point, and then a capillary tube was dipped into the sample container so that the oil rose approximately 1 cm into the capillary tube. The outer surface of the capillary tube containing the sample was cleaned with cotton, and the tube was immediately immersed in an ice water bath to solidify the fat. The samples were then placed at 6°C for 16 h. After this period, the capillary tubes were attached to a thermometer and placed in a 600 mL beaker containing water at a temperature at least 10°C below the expected melting point of the sample. The temperature at which the first upward movement of the fat was observed in the capillary tube was recorded as the SMP of the sample.

#### 2.3.7 pH Measurement

For pH measurement, 5 g of cocoa butter sample was melted at 40°C and mixed with 10 mL of distilled water preheated to 40°C. The mixture was vigorously shaken for 2 min, and the aqueous phase was separated. A pH meter (Metrohm, Switzerland) was used to measure the pH of the aqueous phase after calibrating the device using pH 4 and pH 7 buffers. All measurements were performed in triplicate.

#### 2.3.8 Measurement of Lead Content

To measure the lead content, wet ashing of the sample was first performed. The lead content was determined according to the method described in Iranian National Standard No. 2588, which is based on the Association of Official Analytical Chemists (AOAC) method 999.11 for heavy metal analysis in fats and oils. The method involves wet ashing, acid digestion, and atomic absorption spectrophotometric measurement. For this purpose, 5 g of the sample was mixed with 30 mL of concentrated nitric acid and hydrogen peroxide, placed on a hot plate, and heated until the final volume reached 2–6 mL. During heating, the mixture was gently stirred. Then, 25 mL of concentrated hydrochloric acid was added, and the mixture was heated again until the final volume reached 10 mL. After cooling to room temperature, the solution was transferred to a 50 mL volumetric flask and diluted to volume with distilled water. A blank sample was prepared in the same manner, using distilled water instead of the sample. An atomic absorption spectrophotometer was used to measure the lead content. A 1000 ppm stock solution was purchased from Merck, and the required concentrations for the standard curve were prepared with 1 M nitric acid. After preparing the standard solutions, their absorbance was read by the instrument, and a standard curve was plotted. Based on the standard curve, the absorbance of the samples was measured at a wavelength of 283.4 nm for lead determination, and the lead concentration was obtained from the standard curve.

The limit of detection (LOD) and limit of quantification (LOQ) for the lead determination method were calculated based on the standard deviation of the blank measurements and the slope of the calibration curve. The LOD was 0.005 mg/kg, and the LOQ was 0.015 mg/kg. These values confirm that the reported lead concentrations (ranging from 0.027 to 0.040 mg/kg) are well above the LOQ, and therefore the differences observed among samples are statistically and analytically meaningful.

### 2.4 Statistical Analysis

In this study, two statistical designs, namely the central composite design (CCD) and the factorial design, were employed to investigate the effects of two independent variables, potassium carbonate (at three levels) and ammonium bicarbonate (at three levels), on the chemical properties of cocoa butter. The central composite design, based on the response surface methodology (RSM), was developed using Design‐Expert software version 10 (Table 1). Mathematical models were established to describe the relationships between the independent variables and the dependent variables. The statistical significance level was set at 95% in this section. For the factorial statistical design, data analysis was performed using one‐way analysis of variance (ANOVA), and significant differences between means were compared using Duncan's multiple range test at a 95% confidence level (Table 2). In this section, data analysis was carried out using SPSS software version 22.

**TABLE 2:** List of experiments designed according to the factorial design.

Run	Factor A: K_2_CO_3_ (%)	Factor B: NH_4_HCO_3_ (%)
C1	0	0
C2	3	0
C3	6	0
C4	0	3
C5	0	6
C6	3	6
C7	6	3
C8	3	3
C9	6	6

## 3 Results and Discussion

### 3.1 Response Surface Methodology

Response surface methodology (RSM) is one of the concepts in experimental design. This method is useful for analyzing experiments in which one or more independent variables (responses) are affected by numerous factors, and the objective is to optimize the response. One of the advantages of using this method and Design‐Expert software, in addition to reducing the number of experiments, is the possibility of presenting a mathematical relationship between the independent variables and the dependent variables. Furthermore, this method allows for investigating the effects of qualitative variables alongside quantitative ones. In this study, a central composite design (CCD) was employed to investigate the effects of the independent parameters (potassium carbonate and ammonium bicarbonate) on the dependent responses (acidity, peroxide value, iodine value, saponification value, moisture content, slip melting point, pH, and lead content). Table 3 presents the mathematical models describing the relationships between the independent and dependent variables for cocoa butter. No statistically significant response‐surface model could be developed for the peroxide value, as the variations across treatments were minimal (0.076–0.10 meq O_2_/kg) and did not follow a consistent polynomial trend within the studied concentration range (0%–6%). Therefore, the interpretation of peroxide value is based on the factorial analysis presented in Table 4, which provides a clearer comparison between individual treatments. This observation suggests that the peroxide value is not strongly influenced by the concentration of the alkaline agents under the conditions tested.

**TABLE 3:** Mathematical models and relationships between independent and dependent variables.

Response	Equations	*R* ^2^	*R* ^2^_Adj_	Model significance (*p*)	Lack of fit (*p*)
Acidity (%)	= + 1.48–0.238*A‐0.261*B‐0.024*A*B‐0.023A^2–0.098B^2	0.97	0.95	< 0.001	0.12
Peroxide (meq/kg)	—	—	—	—	—
Sap val (mgKOH/100 g)	= + 195.297 + 2.296*A + 2.034*B	0.87	0.75	0.002	0.08
I val (g/100 g)	=33.038 + 0.867*A‐0.097A^2	0.62	0.59	0.015	0.21
Moisture (%)	= + 0.074–0.004*A‐0.003*B + 0.000*A*B‐0.008*A^2–0.006B^2	0.81	0.78	< 0.001	0.18
Slip point (°C)	=27.910–0.388*A‐0.361*B0.541*A*B	0.76	0.71	0.003	0.14
pH	=8.151 + 1.030*A + 1.006*B‐0.580*A*B	0.92	0.90	< 0.001	0.10
pb val (ppm)	=0.034 + 0.003*A + 0.004*B	0.76	0.71	0.005	0.22

**TABLE 4:** Comparison of means of measured physicochemical properties of cocoa butter.

Sample	K_2_CO_3_ (%)	NH_4_HCO_3_ (%)	Acidity	pH	Sap val (mgKOH/100 g)	I val (g/100 g)	Moisture (%)	Slip point (°C)	Peroxide (meq/kg)	pb (ppm)
C1	0	0	1.77 ± 0.02^h^	5.05 ± 0.01^a^	189.61 ± 1.01^a^	32.62 ± 0.02^b^	0.071 ± 0.001^d^	30.23 ± 0.25^f^	0.08 ± 0.01^ab^	0.03 ± 0.001^a^
C2	3	0	1.60 ± 0.05^g^	7.69 ± 0.01^c^	193.1 ± 0.22^b^	33.49 ± 0.10^c^	0.061 ± 0.001^b^	29.26 ± 0.25^e^	0.076 ± 0.005^a^	0.027 ± 0.000^a^
C3	6	0	1.29 ± 0.02^d^	8.40 ± 0.02^e^	197.14 ± 0.12^d^	34.49 ± 0.09^f^	0.066 ± 0.002^c^	28.00 ± 0.10^d^	0.086 ± 0.005^abc^	0.037 ± 0.002^b^
C4	0	3	1.54 ± 0.02^f^	7.65 ± 0.05^b^	194.15 ± 1.01^bc^	34.08 ± 0.02^e^	0.066 ± 0.002^c^	27.50 ± 0.1^c^	0.093 ± 0.005^bc^	0.029 ± 0.001^a^
C5	0	6	1.30 ± 0.05^d^	8.45 ± 0.02^f^	195.02 ± 0.53^c^	32.42 ± 0.20^a^	0.064 ± 0.003^c^	30.00 ± 0.10^f^	0.083 ± 0.005^ab^	0.036 ± 0.001^b^
C6	3	6	0.97 ± 0.01^b^	9.27 ± 0.02^g^	197.47 ± 1.00^d^	35.17 ± 0.10^h^	0.065 ± 0.000^c^	26.00 ± 0.50^a^	0.10 ± 0.01^c^	0.040 ± 0.002^c^
C7	6	3	1.17 ± 0.01^c^	9.44 ± 0.02^h^	196.59 ± 0.52^d^	33.69 ± 0.10^d^	0.053 ± 0.001^a^	29.51 ± 0.02^e^	0.083 ± 0.005^ab^	0.038 ± 0.002^bc^
C8	3	3	1.42 ± 0.01^e^	8.11 ± 0.01^d^	194.76 ± 0.22^c^	34.92 ± 0.02^g^	0.077 ± 0.001^e^	27.00 ± 0.20^b^	0.093 ± 0.005^bc^	0.036 ± 0.002^b^
C9	6	6	0.77 ± 0.02^a^	9.87 ± 0.01^i^	199.04 ± 0.56^e^	35.91 ± 0.06^i^	0.054 ± 0.001^a^	27.00 ± 0.10^b^	0.10 ± 0.01^c^	0.04 ± 0.001^c^

*Note:* Values are means ± standard deviation (*n* = 3). Means within the same column with different superscript letters are significantly different (*p* < 0.05) according to Duncan’s multiple range test.

As shown in Table 3, the *R* ^2^ values for the mathematical models range from 0.62 (iodine value) to 0.97 (acidity). Models with *R* ^2^ > 0.85 (acidity, pH, moisture) demonstrate good predictive capacity and can be confidently used for optimization within the studied range. However, models with *R* ^2^ < 0.75 (iodine value, saponification value, slip melting point) should be interpreted with caution. These lower *R* ^2^ values indicate that the linear and quadratic terms included in the model do not fully capture the variability in the data, and the equations should be considered primarily as descriptive rather than predictive tools. The adjusted *R* ^2^ values, which account for the number of predictors in the model, are also presented to provide a more conservative estimate of model fit.

The results of data analysis using the factorial statistical method, performed by one‐way analysis of variance (ANOVA) and comparison of significant differences between means (three replicates) using Duncan's multiple range test at a 95% confidence level, are also reported in Table 4.

### 3.2 Acidity and pH

Figure 2 shows the three‐dimensional response surface plot illustrating the effects of potassium carbonate and ammonium bicarbonate on the acidity and pH of cocoa butter. Furthermore, the results of the analysis of variance (ANOVA) for the measured acidity and pH of cocoa butter are reported in Table 4. According to Figure 2 and Table 4, there is a significant difference (*p* < 0.05) between the mean acidity values of the cocoa butter samples and the control sample at the 95% confidence level. This difference is also significant among the alkalized cocoa butter samples. The only non‐significant differences (*p* > 0.05) at the 95% confidence level were observed between the mean acidity of sample C3 (alkalized with 6% potassium carbonate) and sample C5 (alkalized with 6% ammonium bicarbonate). The highest acidity value was recorded for the control sample, while the lowest was observed in sample C9 (alkalized with 6% potassium carbonate and 6% ammonium bicarbonate). According to the results, samples alkalized with 3% potassium carbonate exhibited higher acidity compared to those alkalized with 3% ammonium bicarbonate. At a concentration of 6% of these alkaline agents, the produced samples showed no significant difference in acidity. The application of 6% of either alkaline compound alone had a greater reducing effect on acidity compared to the combined use of 3% potassium carbonate and 3% ammonium bicarbonate for alkalization.

**FIGURE 2:**
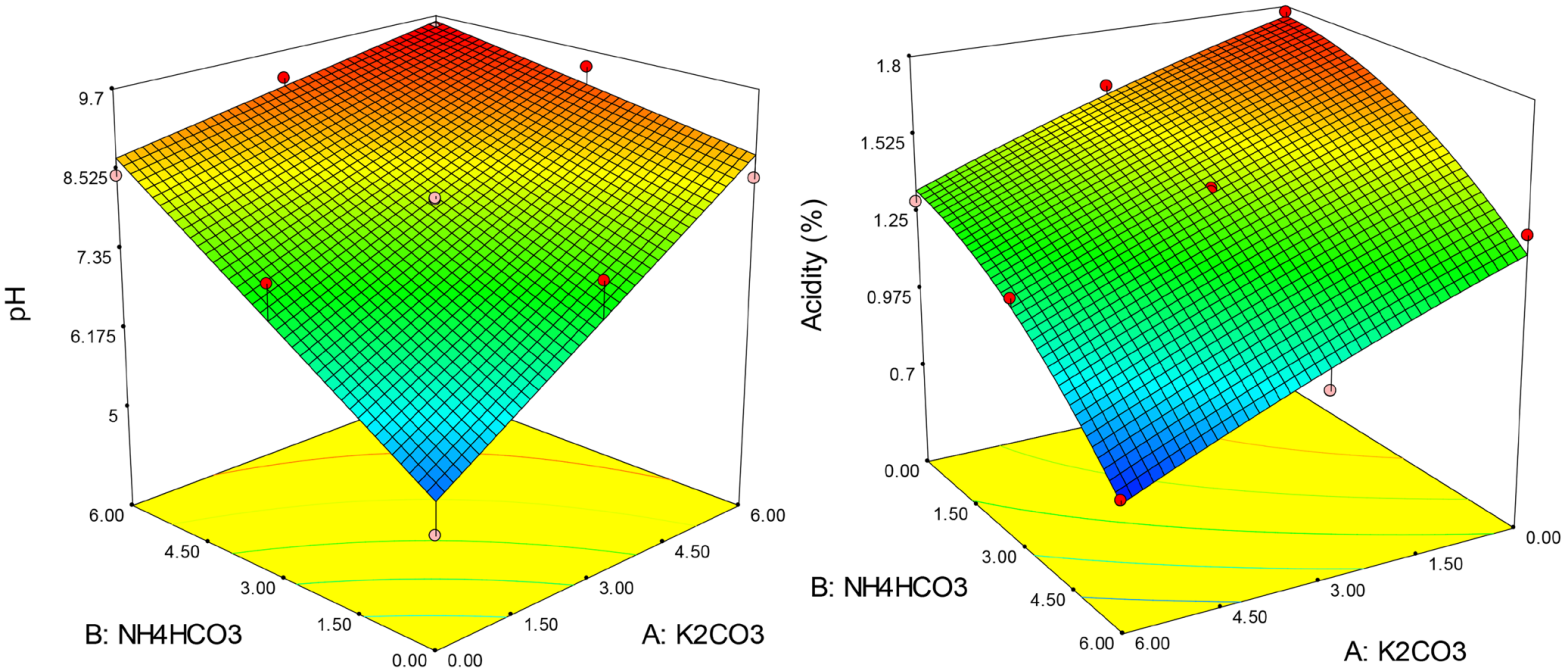
Three‐dimensional response surface plot showing the effects of potassium carbonate and ammonium bicarbonate on the acidity and pH of cocoa butter.

The reduction in acidity upon alkalization can be explained by the neutralization of free fatty acids (FFAs) present in cocoa butter. The alkaline compounds dissociate in water, releasing OH^−^ ions that react with FFAs to form salts (soaps), thereby reducing the measurable acid value (De Leonardis et al. 2016). The higher efficacy of ammonium bicarbonate at lower concentrations may be attributed to its complete dissociation into ammonium (NH_4_^+^) and bicarbonate (HCO_3_^−^) ions, compared to potassium carbonate, which forms carbonate (CO_3_^2−^) ions that require two protonation steps for complete neutralization.

Acidity in oils is a function of the content of free fatty acids (FFAs). In the samples produced in this study, except for the control sample, the alkaline compounds potassium carbonate and ammonium bicarbonate were used. Therefore, the acidity of the samples depends not only on the FFA content but also on the amount and type of alkali used. Considering that the oxidation rate of free fatty acids is higher than that of fatty acids incorporated into the triglyceride structure, it can be stated that the lower the FFA content, the more the acid value decreases, and consequently, the oil will exhibit greater stability (De Leonardis et al. 2016).

The fatty acid composition of oils is not only an important parameter for determining nutritional value, oil applications, and oxidative stability, but it is also a compositional and identification index indicating the purity of various oil types. Oils undergo qualitative changes over time due to various factors such as heat and contamination, and the acid value represents one of the indicators of these changes. During hydrolysis, in addition to an increase in free fatty acids, monoacylglycerols and diacylglycerols are also formed as a result of triacylglycerol hydrolysis (Guillaume et al. 2018). According to Table 4, there is a significant difference (*p* < 0.05) between the mean acidity values of most cocoa butter samples with each other and with the control sample at the 95% confidence level.

The highest acidity was observed in the control sample, while the lowest was found in sample C9 (alkalized with 6% potassium carbonate and 6% ammonium bicarbonate). Due to the use of alkaline agents for processing, the acidity of cocoa butter decreased in the alkalized samples. Samples containing higher amounts of alkali exhibited lower acidity. Among most samples alkalized with ammonium bicarbonate and potassium carbonate, those alkalized with ammonium bicarbonate showed lower acidity compared to cocoa butter samples alkalized with the same concentration of potassium carbonate. This can be attributed to the neutralization of free fatty acids by the alkaline compound used. Although alkalization with 6% potassium carbonate and 6% ammonium bicarbonate resulted in the lowest acidity, this sample had a high peroxide value. In terms of acidity, after this sample, the samples alkalized with 3% potassium carbonate and 6% ammonium bicarbonate exhibited the next lowest acidity. According to Iranian National Standard No. 609 (ISIRI 2010), the maximum allowable acidity of cocoa butter samples, expressed as oleic acid, is 1.75%. All samples examined in this study had normal acidity levels, except for the control sample, which had an acidity of 1.77%, slightly exceeding the standard limit. During the alkalization process, in addition to the presence of alkali, the neutralization of fatty acids also occurs, which itself leads to a reduction in acidity.

Li et al. (2012) studied the effect of alkalization on bioactive components, volatile flavor compounds, and flavor‐related constituents in cocoa powder. The results of that study showed that acetic acid, which was the predominant acid in cocoa powders before alkalization, was significantly reduced (by 67.15%). Sioriki et al. (2021) investigated the effect of the degree of alkalization (medium and high) on aroma‐active compounds in cocoa powder and sensory properties. The results indicated that alkalization reduced the levels of volatile compounds responsible for acidity, fruity, sweet, and cocoa aromas. Osundahunsi et al. (2007) examined the effect of cocoa pod husk ash as an alkalizing agent on cocoa bean products. These researchers reported free fatty acid levels ranging from 1.44% to 1.59%.

According to the three‐dimensional pH plot and the corresponding results in Table 4, there is a significant difference (*p* < 0.05) at the 95% confidence level between the mean pH of the control cocoa butter sample and the alkalized cocoa butter samples, as well as among the alkalized cocoa butter samples themselves. The lowest pH was related to the control sample, while the highest pH was observed in sample C9 (containing 6% potassium carbonate and 6% ammonium bicarbonate). At equal concentrations of each alkaline compound used for alkalization, 3% potassium carbonate and 6% ammonium bicarbonate resulted in higher pH values in the cocoa butter samples. As the concentration of the alkali used for alkalization increased, the pH of the samples increased.

Rodríguez et al. (2009) used sodium carbonate, sodium bicarbonate, and sodium hydroxide, and reported that the pH of cocoa liquor increased from 5 for the control sample to 9.9 for the sample alkalized with 30 g/kg sodium hydroxide. When alkaline compounds are used for cocoa processing, free fatty acids are neutralized, thereby decreasing acidity and increasing pH. Osundahunsi et al. (2007) investigated the effect of cocoa pod husk ash as an alkalizing agent on cocoa bean products. In that study, imported alkaline agents were used to produce alkalized/Dutch‐processed cocoa nibs in the cocoa processing industry. Cocoa powder and cocoa butter were produced from cocoa nibs treated with cocoa pod husk ash as an alkalizing agent and were compared with products from two industries that used imported alkali as the Dutching agent. Cocoa products made from cocoa nibs alkalized with pod husk ash were evaluated for physicochemical properties in comparison with products from Oluji and Stanmark industries located in southwestern Nigeria. The pH values of the cocoa powder were reported as 6.72 and 6.56 for the Oluji and Stanmark samples, respectively, and 6.59 for the tested cocoa powder sample. Miller et al. (2008) investigated the physical and chemical properties of natural and alkalized cocoa powders. As part of that study, brown, black, and red cocoa powders processed with alkali were examined. Natural cocoa powder has a pH range of 5.3–8.9. Alkalized cocoa powders were classified into light treatment (pH 6.50–7.20), medium treatment (pH 7.21–7.60), and heavy treatment (pH 7.61 and above) groups.

### 3.3 Iodine Value and Saponification Value

Figure 3 shows the three‐dimensional response surface plots illustrating the effects of potassium carbonate and ammonium bicarbonate on the saponification value and iodine value of cocoa butter. Furthermore, the results of the analysis of variance (ANOVA) for the measured saponification and iodine values of cocoa butter are reported in Table 4. According to Figure 3 and Table 4, there is a significant difference (*p* < 0.05) at the 95% confidence level between the mean saponification value of the control cocoa butter sample and the alkalized samples. No significant differences (*p* > 0.05) at the 95% confidence level were observed between the mean saponification values of sample C2 (alkalized with 3% potassium carbonate) and sample C4 (alkalized with 3% ammonium bicarbonate), as well as between sample C4 and sample C5 (alkalized with 6% ammonium bicarbonate), and sample C8 (alkalized with 3% potassium carbonate and 3% ammonium bicarbonate). Furthermore, the differences between the saponification values of sample C3 (alkalized with 6% potassium carbonate), sample C6 (alkalized with 3% potassium carbonate and 6% ammonium bicarbonate), and sample C7 (alkalized with 6% potassium carbonate and 3% ammonium bicarbonate) were also not significant (*p* > 0.05) at the 95% confidence level. The lowest saponification value was recorded for the control sample, while the highest was observed in sample C9 (alkalized with 6% potassium carbonate and 6% ammonium bicarbonate). Overall, among the alkalized samples, potassium carbonate at a concentration of 3% and ammonium bicarbonate at a concentration of 6% exhibited lower saponification values compared to the other alkaline compound used at the same concentration. The combined use of 3% potassium carbonate and 3% ammonium bicarbonate resulted in a lower saponification value compared to samples alkalized with 6% of either alkaline compound alone. Therefore, with respect to the saponification value, the combined application of 3% of each alkaline compound yields better results than using 6% of either alkali alone. Samples alkalized with 6% potassium carbonate and 6% ammonium bicarbonate exhibited the highest saponification value, even exceeding the standard limit.

**FIGURE 3:**
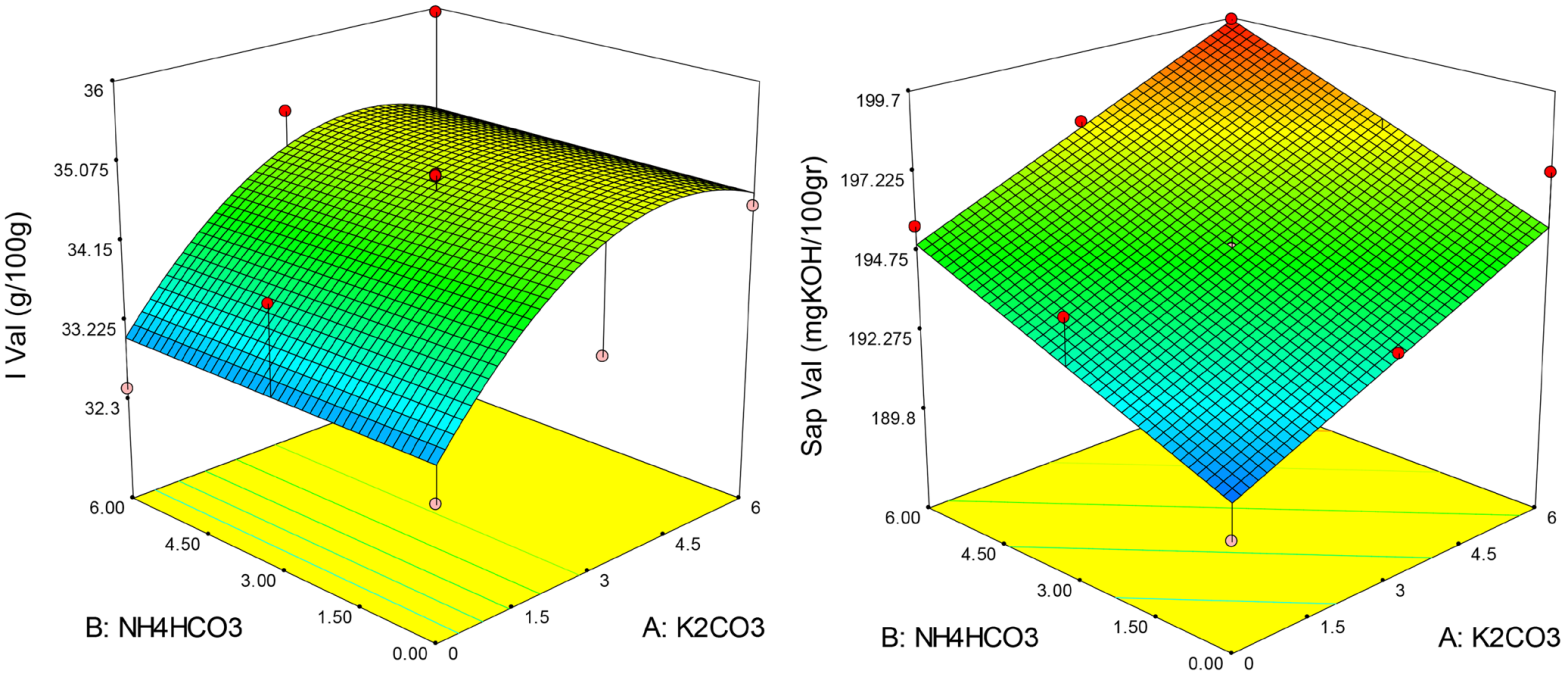
Three‐dimensional response surface plot showing the effects of potassium carbonate and ammonium bicarbonate on the iodine value and saponification value of cocoa butter.

The term saponification refers to the hydrolysis of an ester into an alcohol and an acid. In oils and fats, the reaction between an alkali and fat leads to the formation of soap and glycerol.

The increase in saponification value suggests that alkalization induces some degree of triacylglycerol hydrolysis, releasing shorter‐chain fatty acids that require more KOH per gram for saponification. This is consistent with the observed decrease in melting point (Section 3.4), as shorter‐chain fatty acids generally have lower melting points. The effect is more pronounced with higher alkali concentrations, indicating that hydrolysis is concentration‐dependent. The saponification value of an oil is defined as the number of milligrams of potassium hydroxide required to saponify one gram of oil. Measurement of the saponification value of fats and oils is used to determine the degree of purity of samples, the chemical composition of the fat or oil, and the presence of adulterants. The saponification value is an indicator of the relative molecular weight of the constituent glycerides in the oil. If the triglycerides contain fatty acids with low molecular weights, the number of molecules present in one gram of fat is higher than in cases where the fatty acids have long chains with high molecular weights. Therefore, the lower the molecular weight of the constituent fatty acids of a fat, the higher its saponification value, and conversely, this index can be used to determine the average molecular weight of the fatty acids in a fat. According to Table 4, there is a significant difference (*p* < 0.05) at the 95% confidence level between the mean saponification value of the control cocoa butter sample and the alkalized samples. The lowest saponification value was related to the control sample, while the highest was observed in sample C9 (alkalized with 6% potassium carbonate and 6% ammonium bicarbonate). As evident from the results, the use of higher amounts of the alkaline agents potassium carbonate and ammonium bicarbonate leads to an increase in the saponification value. It is likely that during the alkalization process, the fatty acids undergo some degree of change, acquiring a lower molecular weight; for example, decomposition and depolymerization can lead to a reduction in molecular weight. The sample alkalized with 3% potassium carbonate, followed by the sample alkalized with 3% ammonium bicarbonate, had saponification values closest to that of the control sample. According to Iranian National Standard No. 609, the saponification value of cocoa butter should range between 188 and 198 mg KOH/g oil. All samples examined in this study complied with the Iranian national standard regarding saponification value, except for sample C9, which was alkalized with 6% potassium carbonate and 6% ammonium bicarbonate. Osundahunsi et al. (2007) investigated the effect of cocoa pod husk ash as an alkalizing agent on cocoa bean products. In that study, imported alkaline agents were used to produce alkalized/Dutch‐processed cocoa nibs in the cocoa processing industry. Cocoa powder and cocoa butter were produced from cocoa nibs treated with cocoa pod husk ash as an alkalizing agent and were compared with products from two industries that used imported alkali as the Dutching agent. Cocoa products made from cocoa nibs alkalized with pod husk ash were evaluated for physicochemical properties in comparison with products from Oluji and Stanmark industries located in southwestern Nigeria. According to the results, the saponification value was 193 mg KOH/g for the test sample, while the Stanmark and Oluji cocoa butters had saponification values of 196 and 198 mg KOH/g, respectively.

Regarding the iodine value results shown in Figure 3 and Table 4, there is a significant difference (*p* < 0.05) at the 95% confidence level between the mean iodine value of the control sample and the other samples, as well as among the other samples themselves. The highest iodine value was observed in sample C9 (alkalized with 6% potassium carbonate and 6% ammonium bicarbonate), while the lowest was recorded for sample C5 (alkalized with 6% ammonium bicarbonate). At lower concentrations of potassium carbonate (3%) and higher concentrations of ammonium bicarbonate (6%), the iodine value was higher. When both alkalis were used at their maximum levels (6%), the iodine value was higher. According to the results, the use of 3% potassium carbonate in alkalization had a greater reducing effect on the iodine value compared to 3% ammonium bicarbonate. Furthermore, the use of 6% potassium carbonate in alkalization had a smaller reducing effect on the iodine value compared to 6% ammonium bicarbonate, and the sample alkalized with 6% ammonium bicarbonate had a lower iodine value than the sample alkalized with 6% potassium carbonate. The combined use of 3% ammonium bicarbonate and 3% potassium carbonate had an effect similar to that of using 6% potassium carbonate alone. Overall, among the alkalized cocoa butter samples, the lowest iodine value was associated with the sample alkalized with 6% ammonium bicarbonate.

The iodine value is defined as the weight of iodine, in grams, that is fixed by 100 g of a fatty substance under specific conditions. In other words, it is the amount of iodine absorbed by 100 g of a fatty substance. This value depends on the degree of unsaturation of the fatty acids in the fatty substance. There is a significant difference (*p* < 0.05) at the 95% confidence level between the mean iodine value of the control sample and the other samples, as well as among the other samples themselves. The highest iodine value was related to sample C9 (alkalized with 6% potassium carbonate and 6% ammonium bicarbonate), while the lowest was associated with sample C5 (alkalized with 6% ammonium bicarbonate). The sample alkalized with 6% ammonium bicarbonate, followed by the sample alkalized with 3% potassium carbonate, had iodine values closer to that of the control sample. At lower concentrations of potassium carbonate (3%) and higher concentrations of ammonium bicarbonate (6%), the iodine value was higher. When both alkalis were used at their maximum levels (6%), the iodine value was higher. The high iodine value in samples alkalized with 6% potassium carbonate and 6% ammonium bicarbonate indicates a higher content of unsaturated fatty acids. It is likely that alkalization does not have a negative effect on reducing unsaturated fatty acids. On the other hand, the peroxide value of these samples was also higher, indicating that a higher degree of unsaturation increases the rate of autoxidation in oils and fats. According to Iranian National Standard No. 609, the iodine value should be in the range between 32 and 42. All samples examined in this test complied with the Iranian national standard regarding iodine value. Osundahunsi et al. (2007) investigated the effect of cocoa pod husk ash as an alkalizing agent on cocoa bean products. In that study, the iodine values of the samples ranged between 35.11 and 38.07, which is consistent with the results of the present study.

### 3.4 Moisture Content and Slip Melting Point

Figure 4 shows the three‐dimensional response surface plots illustrating the effects of potassium carbonate and ammonium bicarbonate on the moisture content and slip melting point of cocoa butter. Furthermore, the results of the analysis of variance (ANOVA) for the measured moisture content of cocoa butter are reported in Table 4. According to the results in Figure 4 and Table 4, there is a significant difference (*p* < 0.05) at the 95% confidence level between the mean moisture content of the control sample and the other alkalized cocoa butter samples. No significant differences (*p* > 0.05) at the 95% confidence level were observed between the mean moisture content of sample C3 (alkalized with 6% potassium carbonate) and samples C4 (alkalized with 3% ammonium bicarbonate), C5 (alkalized with 6% ammonium bicarbonate), and C6 (alkalized with 3% potassium carbonate and 6% ammonium bicarbonate). Furthermore, there was no statistically significant difference (*p* > 0.05) at the 95% confidence level between the mean moisture content of sample C7 (alkalized with 6% potassium carbonate and 3% ammonium bicarbonate) and sample C9 (alkalized with 6% potassium carbonate and 6% ammonium bicarbonate). The highest moisture content was observed in sample C8 (alkalized with 3% potassium carbonate and 3% ammonium bicarbonate), while the lowest was recorded for sample C7 (alkalized with 6% potassium carbonate and 3% ammonium bicarbonate). Among the alkalized samples, those alkalized with 3% potassium carbonate had lower moisture content compared to those alkalized with 3% ammonium bicarbonate, while samples alkalized with 6% potassium carbonate and those alkalized with 6% ammonium bicarbonate exhibited similar moisture contents. The sample alkalized with 3% potassium carbonate and 3% ammonium bicarbonate had higher moisture content compared to samples alkalized with 6% of either alkaline compound alone.

**FIGURE 4:**
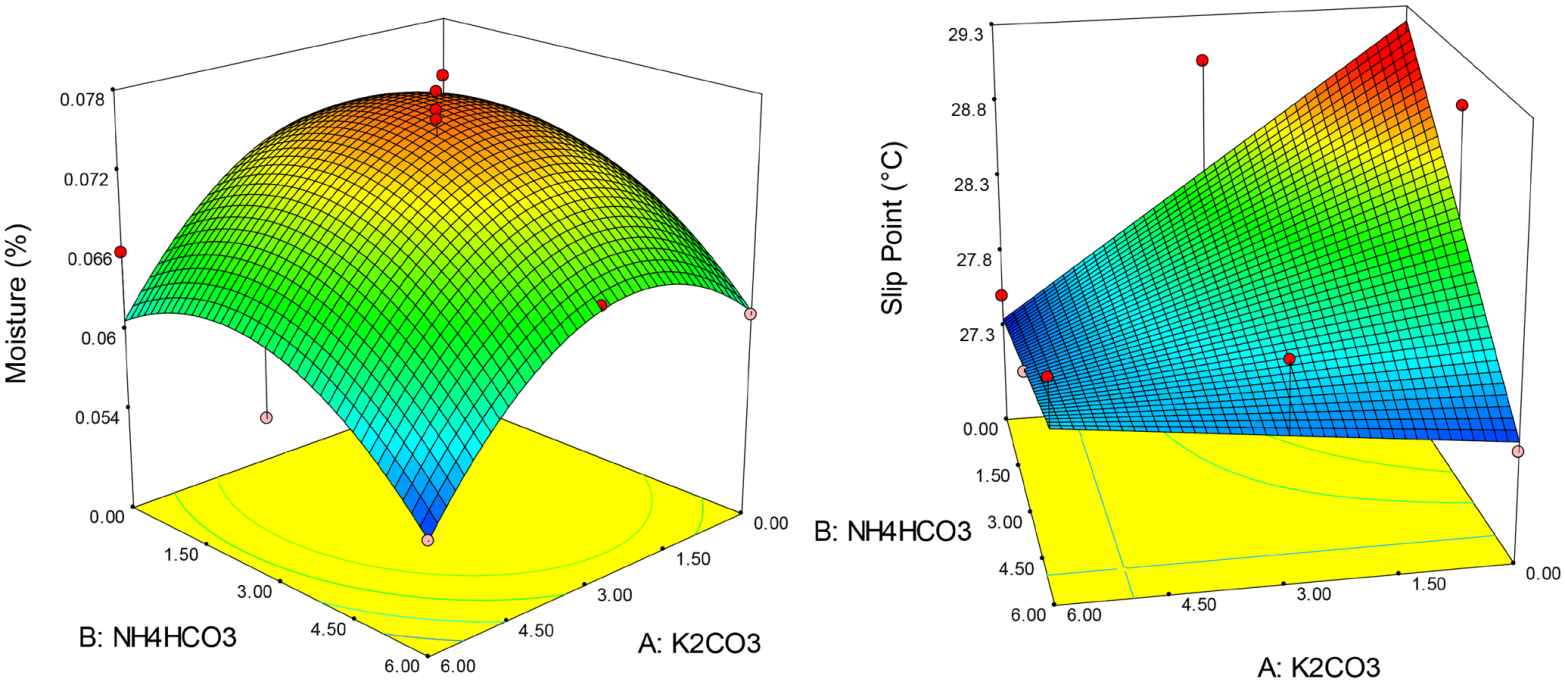
Three‐dimensional response surface plot showing the effects of potassium carbonate and ammonium bicarbonate on the moisture content and slip melting point of cocoa butter.

The presence of moisture in the sample accelerates the hydrolysis rate of triglycerides. Hydrolysis of triglycerides occurs in the presence of water and with the aid of the lipase enzyme, increasing the free fatty acid content. According to the results in Table 4, there is a significant difference (*p* < 0.05) at the 95% confidence level between the mean moisture content of the control sample and the other alkalized cocoa butter samples. The highest moisture content was related to sample C8 (alkalized with 3% potassium carbonate and 3% ammonium bicarbonate), while the lowest was associated with sample C7 (alkalized with 6% potassium carbonate and 3% ammonium bicarbonate). Alkalization of samples with 12% alkaline agents (6% potassium carbonate and 6% ammonium bicarbonate) resulted in the lowest moisture content. According to the acidity results obtained, these samples exhibited the lowest acidity, which is consistent with the moisture content present in the samples. According to Iranian National Standard No. 609, the maximum moisture content in cocoa butter samples is 0.1%. All examined samples complied with the standard regarding moisture percentage. Since moisture increases the rate of hydrolytic rancidity reactions in fat and oil samples, cocoa butter samples with higher moisture content undergo more hydrolysis, resulting in higher acidity due to increased free fatty acids.

Regarding the melting point results shown in Figure 4 and Table 4, there is a significant difference (*p* < 0.05) at the 95% confidence level between the mean melting point of the control cocoa butter sample and the alkalized samples. The only non‐significant difference (*p* > 0.05) at the 95% confidence level was observed between the control sample and the alkalized sample C5 (alkalized with 6% ammonium bicarbonate). There are significant differences among the mean slip melting points of the alkalized samples at the 95% confidence level, except between sample C2 (alkalized with 3% potassium carbonate) and sample C7 (alkalized with 6% potassium carbonate and 3% ammonium bicarbonate), as well as between sample C8 (alkalized with 3% potassium carbonate and 3% ammonium bicarbonate) and sample C9 (alkalized with 6% potassium carbonate and 6% ammonium bicarbonate), where no significant differences (*p* > 0.05) were observed. The highest melting point was related to the control sample, while the lowest was observed in sample C6 (alkalized with 3% potassium carbonate and 6% ammonium bicarbonate). Samples alkalized with 3% potassium carbonate exhibited higher slip melting points compared to those alkalized with 3% ammonium bicarbonate. Cocoa butter samples alkalized with 6% potassium carbonate had lower melting points compared to those alkalized with 6% ammonium bicarbonate. In samples alkalized with equal ratios of the two alkalis (3% or 6%), there was no significant difference in slip melting point.

Oils and fats do not possess a well‐defined melting point because fatty materials are composed of a mixture of different glycerides, each having a specific melting point. Upon heating, glycerides melt sequentially until a temperature is reached at which all glycerides have melted and the oil becomes completely liquefied. This melting point is achieved gradually and cannot be a sharp, well‐defined point. According to the results obtained from this study, there is a significant difference (*p* < 0.05) at the 95% confidence level between the mean melting point of the control cocoa butter sample and the alkalized samples. The only non‐significant difference at the 95% confidence level was observed between the control sample and the alkalized sample C5 (alkalized with 6% ammonium bicarbonate). The highest melting point was related to the control sample, while the lowest was observed in sample C6 (alkalized with 3% potassium carbonate and 6% ammonium bicarbonate). The sample alkalized with 6% ammonium bicarbonate had a melting point close to that of the control sample. Following this sample, the sample alkalized with 6% potassium carbonate and 3% ammonium bicarbonate, as well as the sample alkalized with 3% potassium carbonate, exhibited melting points close to that of the control sample. The melting point of saturated fatty acids is higher than that of unsaturated fatty acids. Therefore, if the content of unsaturated fatty acids in an oil or fat is high, its melting point will be lower. Thus, in the cocoa butter samples examined in this study, samples containing higher levels of unsaturated fatty acids (and consequently having lower iodine values) exhibited lower melting points. The control sample had the highest melting point and a lower iodine value compared to most alkalized samples, while sample C6, which had the lowest melting point, exhibited a higher iodine value than most of the examined samples. Deora et al. (2013) stated that the melting point of cocoa butter varies depending on its crystal form (polymorphic forms). According to their results, the melting point of cocoa butter was 16°C–18°C for the β′ form, 21°C–22°C for the α form, 25.5°C for the mixed form, 27°C–29°C for the β_1_ form, 34°C–35°C for the β_2_ form, and 36°C for the β′ form. In the present study, with increasing concentration of the alkaline agent potassium carbonate, as well as the mixture of potassium carbonate and ammonium bicarbonate, the melting point decreased. The results of the present study are consistent with those of Sulistyowati and Misnawi (2008). These researchers used alkaline agents for cocoa alkalization and reported that with increasing alkali concentration, the melting point increased, and with decreasing alkali concentration, the melting point decreased. Of course, in that study, the alkalization temperature was also investigated.

### 3.5 Peroxide Value and Lead Content

Figure 5 shows the three‐dimensional response surface plots illustrating the effects of potassium carbonate and ammonium bicarbonate on the peroxide value and lead content of cocoa butter. The results of the analysis of variance (ANOVA) for the measured peroxide value of cocoa butter are reported in Table 4. According to the results presented in Figure 5 and Table 4, there is a statistically significant difference (*p* < 0.05) at the 95% confidence level between the mean peroxide value of the control sample and samples C6 (alkalized with 3% potassium carbonate and 6% ammonium bicarbonate) and C9 (alkalized with 6% potassium carbonate and 6% ammonium bicarbonate). However, the differences between these two samples (C6 and C9) and sample C8 (alkalized with 3% potassium carbonate and 3% ammonium bicarbonate), sample C4 (alkalized with 3% ammonium bicarbonate), and sample C3 (alkalized with 6% potassium carbonate) are not significant (*p* > 0.05). The highest peroxide values were observed in samples C9 and C6, while the lowest peroxide value was recorded for sample C2 (alkalized with 3% potassium carbonate). According to the results, the use of 3% potassium carbonate in alkalization had a greater reducing effect on the peroxide value compared to 3% ammonium bicarbonate. The use of 6% potassium carbonate in alkalization did not show a statistically significant effect compared to 3% ammonium bicarbonate. The combined use of 3% ammonium bicarbonate and 3% potassium carbonate had an effect similar to that of using 3% ammonium bicarbonate alone. Overall, among the alkalized cocoa butter samples, the lowest peroxide value was associated with the sample alkalized with 3% potassium carbonate.

**FIGURE 5:**
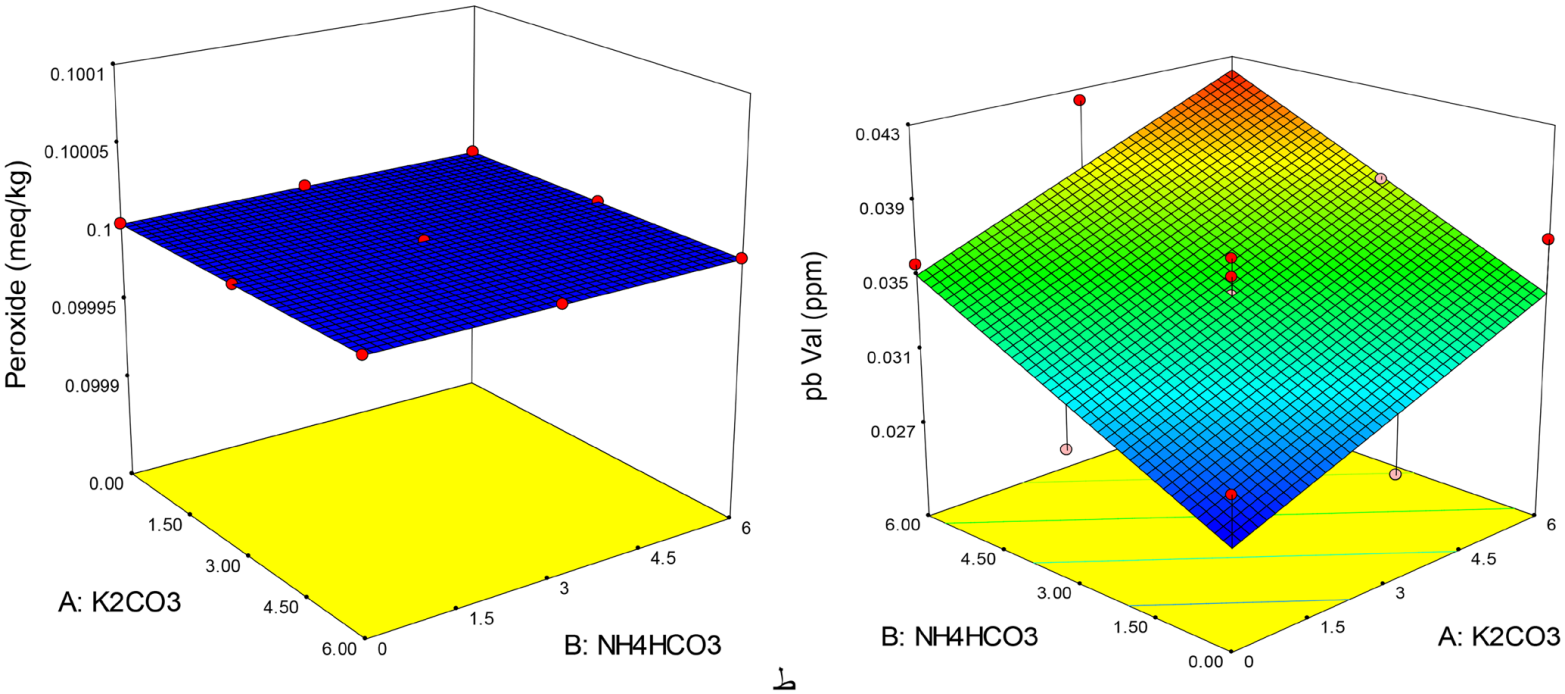
Three‐dimensional response surface plot showing the effects of potassium carbonate and ammonium bicarbonate on the peroxide value and lead content of cocoa butter.

At low temperatures, the oxidation of fatty acids is mainly related to the formation of hydroperoxides, in which case unsaturated compounds are not reduced; however, at high temperatures, a large number of double bonds become saturated. Oils that contain a greater number of double or multiple bonds oxidize more rapidly under the same conditions and are more susceptible to oxidative rancidity. For this reason, the oxidative stability of oils at high temperatures is of great importance. One important and common characteristic of many vegetable oils is their high content of unsaturated fatty acids, which makes them susceptible to oxidative deterioration. Peroxide is the primary product of fat oxidation, and in general, the higher the degree of unsaturation of an oil, the greater its susceptibility to oxidation. The peroxide values obtained in this study (0.076–0.10 meq O_2_/kg) are remarkably low compared to typical values reported in literature for edible oils. This indicates that the cocoa butter used in this study was of high quality and exhibited excellent oxidative stability. All samples remained well below the Iranian standard maximum of 1 meq O_2_/kg, suggesting that the alkalization process, even at high concentrations, did not induce significant primary oxidation. The slightly higher peroxide values observed in samples with high alkali concentrations (C6 and C9) may be explained by the pro‐oxidant effect of metal ions present as impurities in the alkaline agents, although the absolute increase was minimal. Importantly, the low peroxide values across all treatments confirm that the processing conditions employed did not compromise the oxidative quality of the cocoa butter.

When the peroxide value reaches a certain level, various changes occur, and volatile aldehyde and ketone compounds, as well as short‐chain fatty acids, are formed, which contribute to the unpleasant odor and taste of the fatty substance. Although the peroxide formed does not directly cause unpleasant odors and tastes in oils and fats, it is an indicator of the progress of oxidation. Peroxide formation occurs very slowly in the initial stages, and this period may vary from several days to several months depending on the type of oil, storage conditions, temperature, etc. After that, peroxide formation accelerates and acts as a catalyst in accelerating oil oxidation. In addition to peroxides, hydroperoxides are also among the primary oxidation products. Hydroperoxides are tasteless and odorless but decompose rapidly, producing aldehydes and ketones that have unpleasant odors and tastes (Samakradhamrongthai 2024).

Hydroperoxide formation is an autocatalytic reaction, and approximately 90% of the resulting hydroperoxides are in conjugated and trans forms. The amount of trans hydroperoxides increases with increasing temperature. The peroxide value alone cannot provide a complete assessment of fat quality because peroxides are unstable; thus, the peroxide value increases to a certain point and then decreases due to decomposition during extended storage or processing (Kontogianni and Gerothanassis 2022).

According to the results presented in Table 4, there is a statistically significant difference (*p* < 0.05) at the 95% confidence level between the mean peroxide value of the control sample and samples C6 (alkalized with 3% potassium carbonate and 6% ammonium bicarbonate) and C9 (alkalized with 6% potassium carbonate and 6% ammonium bicarbonate). The highest peroxide values were related to these two samples, while the lowest peroxide value was observed in sample C2 (alkalized with 3% potassium carbonate). Alkalization with 3% potassium carbonate resulted in a lower peroxide value compared to the control sample. The remaining samples had higher peroxide values than the control sample. After this sample, samples alkalized with 6% ammonium bicarbonate, as well as the combination of 6% ammonium bicarbonate and 3% potassium carbonate, exhibited lower peroxide values. It is possible that the presence of ionized alkaline compounds can partially inhibit oxidation. Osundahunsi et al. (2007) investigated the effect of cocoa pod husk ash as an alkalizing agent on cocoa bean products. In that study, imported alkaline agents were used to produce alkalized/Dutch‐processed cocoa nibs in the cocoa processing industry. According to their results, the peroxide value varied between 26 and 29 meq O_2_/kg. These results are much higher than the Iranian national standard limit. According to Iranian National Standard No. 609, the maximum allowable peroxide value of cocoa butter samples, expressed as oleic acid, is 1 meq O_2_/kg of fat.

Regarding the lead content results shown in Figure 5 and Table 4, there is no significant difference (*p* > 0.05) at the 95% confidence level between the mean lead content of the control sample and cocoa butter samples C2 (alkalized with 3% potassium carbonate) and C4 (alkalized with 3% ammonium bicarbonate). However, there is a significant difference (*p* < 0.05) at the 95% confidence level between the mean lead content of the control sample and the other alkalized cocoa butter samples. No significant differences (*p* > 0.05) at the 95% confidence level were observed between the mean lead content of sample C3 (alkalized with 6% potassium carbonate), C5 (alkalized with 6% ammonium bicarbonate), sample C7 (alkalized with 6% potassium carbonate and 3% ammonium bicarbonate), and sample C8 (alkalized with 3% potassium carbonate and 3% ammonium bicarbonate). Furthermore, no significant differences (*p* > 0.05) at the 95% confidence level were observed between the lead content of samples C6 (alkalized with 3% potassium carbonate and 6% ammonium bicarbonate), sample C7 (alkalized with 6% potassium carbonate and 3% ammonium bicarbonate), and sample C9 (alkalized with 6% potassium carbonate and 6% ammonium bicarbonate). The lead content of the samples ranged from 0.027 to 0.040 mg/kg.

According to the results presented in Table 4, there is no significant difference (*p* > 0.05) at the 95% confidence level between the mean lead content of the control sample and cocoa butter samples C2 (alkalized with 3% potassium carbonate) and C4 (alkalized with 3% ammonium bicarbonate). However, there is a significant difference (*p* < 0.05) at the 95% confidence level between the mean lead content of the control sample and the other alkalized cocoa butter samples. The lead content of the samples ranged from 0.027 to 0.040 mg/kg. An important decision‐making factor in evaluating the quality of edible oils and fats is the composition of heavy metals. It has been established that heavy metals affect the oxidation rate of oils, nutritional value, storage properties, and stability. Thus, the determination of heavy metals in vegetable oils is very important (Farzin and Moassesi 2014). Heavy metals are toxic elements that can enter food and affect the health of individuals who consume contaminated foods. The levels of heavy metals in food are an important factor for environmental contamination and have attracted worldwide attention due to their toxic effects, even at very low concentrations. The presence of metals in vegetable oils is attributed to endogenous factors related to plant metabolism as well as exogenous factors due to contamination during agronomic practices, seed production and collection, extraction processes, oil treatment, and packaging and storage systems (Farzin and Moassesi 2014). The presence of metals in vegetable oils depends on many factors. They may originate from soil, fertilizers, etc., and become incorporated into the oil. They may also arise during the production process through operations such as bleaching, refining, and deodorization, or through contamination from metal processing equipment, and consequently remain suspended in the oil. Extremely low levels of these metal ions have adverse effects on color and odor. The presence of metals in the final refined oil is undesirable because metals can facilitate oxidative degradation of the oil and reduce its shelf life. Factors that further influence the oxidation rate include the degree of unsaturation, oxygen concentration, temperature, light, and the presence of metals (primarily transition metals such as iron and copper). Trace metals increase the oxidation rate of edible oils by enhancing the production of free radicals from fatty acids or hydroperoxides (Choe et al. 2019).

It has been established that some living organisms have the ability to uptake and accumulate specific elements in their structure, particularly metals, at high concentrations. The presence of trace metals is an important factor concerning edible oil quality. Heavy metals such as lead cause acute and chronic poisoning, adverse effects on the liver, heart, kidneys, and the immune system. Lead is a natural element and a common industrial metal that is widely distributed in air, water, soil, and food. Lead has adverse health effects even at low levels and can cross the placenta, damaging the developing fetal nervous system (Mandal et al. 2023). Lead poisoning has been reported to be associated with neurological problems, renal dysfunction, and anemia. According to Iranian National Standard No. 609, the maximum allowable lead content in cocoa butter is 0.1 mg/kg of fat. All examined samples complied with the Iranian national standard regarding lead content. The Codex Alimentarius international standard also states that the maximum allowable lead content in cocoa butter samples is 100 ng/g (Anyimah‐Ackah et al. 2019).

## 4 Conclusion

In the present study, the effect of the cocoa butter alkalization process using different concentrations of potassium carbonate and ammonium bicarbonate (ranging from 0% to 6%) on physicochemical properties including acidity, peroxide value, saponification value, iodine value, moisture content, melting point, pH, and lead content was investigated. The results indicated that the application of alkaline treatments significantly (at a 95% confidence level) reduced acidity and melting point while increasing the saponification value, iodine value, and pH compared to the control sample. The greatest reduction in acidity was observed in the sample containing 6% of both alkaline compounds, and the highest pH increase was also recorded in this same sample. Furthermore, the lowest melting point was recorded in the sample treated with 3% potassium carbonate and 6% ammonium bicarbonate. On the other hand, the peroxide value and moisture content also varied depending on the type and concentration of the alkaline agents, such that the highest peroxide values were observed in samples containing high concentrations of both compounds. The analysis of lead content in all samples showed that the measured values were within the permissible limits of the Iranian national standard, confirming the safety of the product in this regard. Based on the overall findings, the use of 3% potassium carbonate alone, 3% ammonium bicarbonate alone, as well as the combination of 3% potassium carbonate with 6% ammonium bicarbonate, are proposed as the most suitable treatments for achieving desirable physicochemical properties in alkalized cocoa butter. However, the findings are considered preliminary, as they are based on physicochemical analyses that, while informative, do not provide a complete picture of the structural and compositional changes induced by alkalization. For a comprehensive understanding of the effects, complementary analyses such as fatty acid profile, triacylglycerol (TAG) composition, differential scanning calorimetry (DSC), and crystallization behavior should be conducted in future work. Finally, using the response surface methodology, valid mathematical models were developed that are capable of establishing a quantitative relationship between the concentration of alkaline agents and the chemical properties of the product. These models enable the prediction and precise adjustment of the alkalizing agent composition in order to achieve the desired characteristics in cocoa butter.

## Author Contributions

**Afghan Sadeghzadeh:** conceptualization, investigation, funding acquisition, methodology. **Maryam Taghinezhad:** conceptualization, methodology, data curation. **Sajad Pirsa:** funding acquisition, writing – original draft, writing – review and editing, visualization, project administration, resources, supervision.

## Funding

The authors have nothing to report.

## Conflicts of Interest

The authors declare no conflicts of interest.

## Data Availability

The data that support the findings of this study are available from the corresponding author upon reasonable request.
